# Effects of vitamin D supplementation in endometriosis: a systematic review

**DOI:** 10.1186/s12958-022-01051-9

**Published:** 2022-12-28

**Authors:** Dimitrios Rafail Kalaitzopoulos, Nicolas Samartzis, Angelos Daniilidis, Brigitte Leeners, Sofia Makieva, Konstantinos Nirgianakis, Ioannis Dedes, Julian Matthias Metzler, Patrick Imesch, Ioannis G. Lempesis

**Affiliations:** 1 Department of Gynecology, University Hospital Zurich, Frauenklinikstr. 10, 8091 Zurich, CH Switzerland; 2 Division of Reproductive Endocrinology, University Hospital Zurich, Frauenklinikstr. 10, 8091 Zurich, CH Switzerland; 3 Department of Gynecology and Obstetrics, Cantonal Hospital Schaffhausen, Geissbergstrasse 81, 8208 Schaffhausen, Switzerland; 4 Department of Obstetrics and Gynecology, 2Nd University, Hippokratio General Hospital, Aristotle University of Thessaloniki, Thessaloniki, Greece; 5 Department of Obstetrics and Gynecology, University Hospital and University of Bern, Friedbühlstrasse 19, 3010 Bern, Switzerland; 6 College of Medical and Dental Sciences, Institute of Metabolism and Systems Research (IMSR), University of Birmingham, Birmingham, UK; 7 Centre for Endocrinology, Diabetes and Metabolism (CEDAM), Birmingham Health Partners, Birmingham, UK; 8 Department of Human Biology, School of Nutrition and Translational Research in Metabolism (NUTRIM), Maastricht University, Maastricht, the Netherlands

**Keywords:** Endometriosis, Vitamin D, 1,25(OH)2D, 25(OH)D, 25(ΟΗ)D3, Supplementation

## Abstract

**Background:**

There is a growing body of human, animal and *in vitro* studies on vitamin D (vit D) substitution in endometriosis. The aim of this systematic review is to critically appraise and qualitatively synthesize the results of the available studies that examine the supplementation of vit D for endometriosis treatment.

**Methods:**

A systematic search of the literature was conducted in four electronic databases (Medline, Cochrane, Scopus, Embase) and grey literature for original research articles on humans, animals and *in vitro* models published in any language.

**Results:**

Four human studies, four animal studies and four *in vitro* studies were included. Quantitative synthesis of human studies showed no significant effect of vit D intake for dysmenorrhea (2 studies, 44 vit D vs 44 placebo, mean -0.71, 95% CI -1.94, 0.51) and non-cyclic pelvic pain (2 studies, 42 vit D vs 38 placebo, mean 0.34, 95% CI -0.02, 0.71). Regarding reproductive outcomes in women with endometriosis after *in vitro* fertilization, the only available study showed no differences between women taking vit D and women taking placebo. Three of the four included animal studies showed regression of endometriotic implants when treated with vit D. The *in vitro* studies demonstrated that vit D decreases invasion and proliferation of endometriotic lesions without affecting apoptosis.

**Conclusions:**

Although *in vitro* and animal studies suggest regression of the endometriotic implants and decrease of invasion and proliferation after vit D supplementation, this was not reflected in the results of the meta-analysis, which showed no benefit of vit D supplementation in patients with endometriosis and dysmenorrhea or non-cyclic pelvic pain as well as on the outcome of IVF treatment. However, given the heterogeneity and the diversity of the available studies, more research is required to shed light on the role of vit D supplementation in women with endometriosis.

**Supplementary Information:**

The online version contains supplementary material available at 10.1186/s12958-022-01051-9.

## Introduction

Endometriosis is an oestrogen-dependent chronic inflammatory condition, characterized by endometrium-like lesions present outside the uterine cavity [1–3]. It mainly affects women of reproductive age, with a referred prevalence of 5 to 10% [2]. Classic symptoms include dysmenorrhea, dyspareunia, chronic pelvic pain, and infertility [2, 3]. The multifactorial aetiology of endometriosis has not yet been completely elucidated; the broad spectrum of multiple disease subtypes (peritoneal endometriosis, ovarian endometrioma, deep infiltrating endometriosis) could partially explain the discrepancies in clinical manifestations and nebulous pathophysiology [3–5]. Nevertheless, genetics, environmental factors, immunity and chronic inflammation have been found to be involved in its pathogenesis [1–3]. Many studies have reported increased inflammatory cytokines, neutrophils, macrophages, and tumour necrosis factor-a in peritoneal fluid [1, 6–8]. Variations in the mechanisms of inflammation could be the reason for the discrepancies concerning pain and infertility between the major subtypes of endometriosis [1]. Among the factors altering or affecting inflammation, vitamin D (vit D), has been examined in several studies in relation to endometriosis [1, 9]. Vit D sufficiency is defined as a circulating concentration > 30–40 ng/ml, insufficiency when the concentration is 20–30 ng/ml and deficiency at < 20 ng/ml. Whether the concentration of vit D (25-hydroxyvitamin-D3) is correlated with the disease and its severity is a subject of ongoing debate with studies exhibiting positive and negative associations [10–13].

**Fig. 1:**
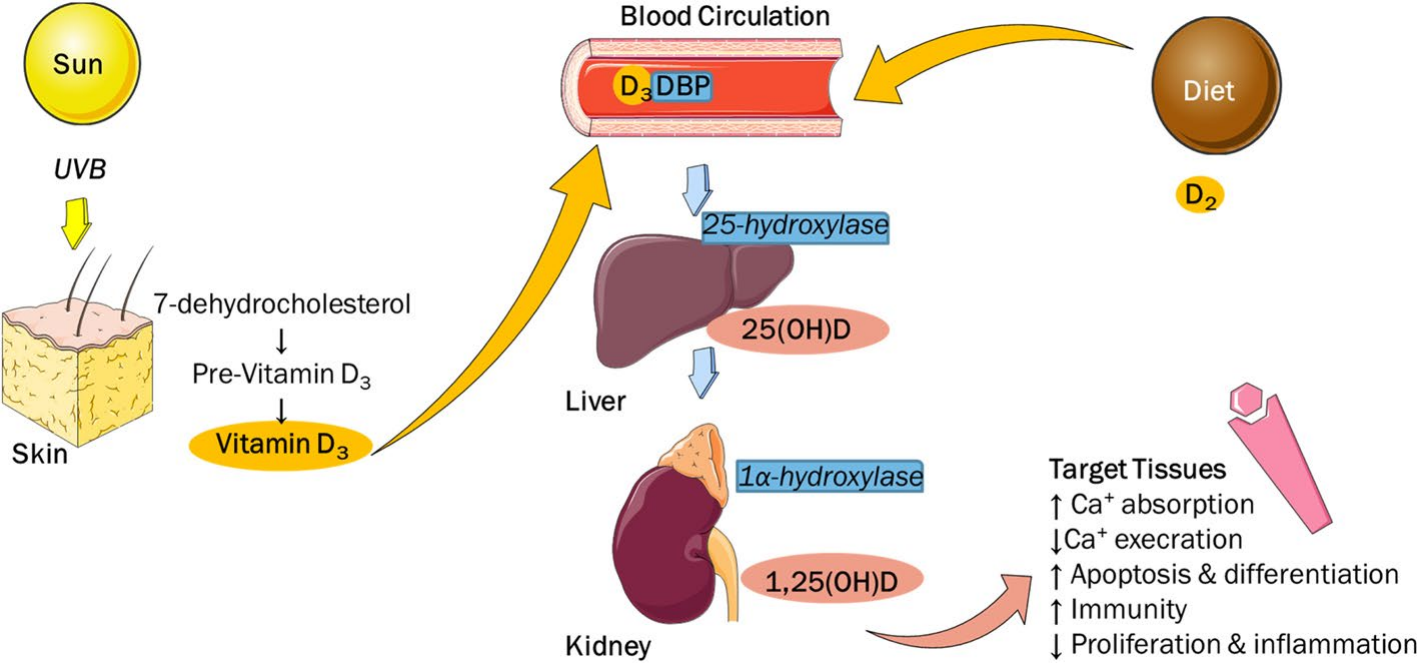
Vitamin D metabolic pathway. Figure’s images source: smart.servier.com Servier Medical Art by Servier is licensed under a Creative Commons Attribution 3.0 Unported License. UVB: ultraviolet B (UVB) rays. DBP: Vitamin D Binding Protein

Vit D’s canonical role is to regulate calcium and skeletal homeostasis, but it has also been shown to be involved in the modulation of the immune system [14]. The vit D metabolic pathway is depicted in Fig. 1 [15]. Most of the biologic actions of vit D are mediated by a high-affinity receptor acting as a transcription factor. The coding gene of vit D receptor (VDR) is located on chromosome 12 [16]. Vienonen et al*. *[17] first reported the expression of VDR protein in the healthy human endometrium of older women with Vigano’ et al*.* [18] confirming later the expression in the younger cyclic endometrium. The presence of VDR in endometrium of patients with endometriosis has also been demonstrated [19]. Although VDR protein is in higher abundance in ectopic endometrium of endometriosis patients compared to controls, this study did not examine the expression in endometriotic lesions [19]. Recently, the expression of VDR has also been demonstrated in peritoneal endometriotic lesions [20]. Currently, it is unclear whether elevated endometrial VDR expression is a primary event or a consequence of endometriosis-associated inflammation. Various immune cells found in endometriosis lesions and known to maintain the disease, have been shown to express VDR and exhibit an active vit D metabolism in other systems [21]. VDR signalling may be an elusive protagonist in the pathophysiology of endometriosis creating opportunities for the development of novel therapeutic targets. The concept of vit D as an anti-endometriosis agent could therefore be considered in the course of disease treatment [21].

The aim of this systematic review was to critically appraise and qualitatively synthesize the results of individual studies that have examined the supplementation of vit D, or relative molecules, in various levels for endometriosis treatment including *in vivo* or *in vitro* models and clinical trials.

## Material and methods

This systematic review was conducted according to the Preferred Reporting Items for Systematic Reviews and Meta-Analyses (PRISMA-P Statement) and registered in PROSPERO (CRD42021265619).

### Search criteria

Medline, Cochrane, Scopus and Embase databases were searched independently by two reviewers (DRK and IGL) until 01.11.2021. The search algorithm included combinations of the terms: vitamin D; calcitriol; cholecalciferol; endometriosis; calcium; 25(OH)D3; 25(OH)D; 1, 25(OH)2D3; vitamin D binding protein; vitamin D receptor. In addition, the references of the included studies were searched manually to identify additional publications potentially missed by the original search.

### Inclusion and exclusion criteria

Original interventional cohort studies and RCTs with vit D supplementation in women diagnosed with endometriosis, animal models with induced endometriosis or *in vitro* experimental models which were published in any language until 01.11.2021 were included. Studies without supplementation of vit D, studies with dysmenorrhea or dyspareunia without histological diagnosis of endometriosis, conference abstracts, case reports and reviews were excluded.

### Outcomes

The primary outcomes for the included human studies were changes in the level of endometriosis associated pain, both dysmenorrhea and non-cyclic pelvic pain, measured in a numerical Visual Analogue Scale (VAS) from 0 to 10 and the cumulative pregnancy rate. Secondary outcomes for the above studies were changes in dyspareunia and dyschezia, also measured according to a numerical analogue scale from 0 to 10.Animal and *in vitro* studies were evaluated for changes of biomarkers and endometriosis relevant cells after vit D treatment.

### Data extraction

Relevant publications were screened independently by two reviewers (DRK, IGL) and data was extracted for each study in a standardized extraction form in an Excel spreadsheet. Where appropriate, the data set was completed through communication with the authors. Specifically, an e-mail was sent and when no answer was received, a second one followed after a two-week interval. Disagreement was resolved by consensus.

### Quality assessment

Two investigators performed the risk of bias assessment of the included studies (DRK and IGL) and any discrepancies were resolved via consensus. Human studies were assessed with Cochrane risk-of-bias tool for randomized studies [22] and animal studies with SYRCLE’s risk of bias tool [23]. For *in vitro* studies no standardized quality assessment tool exists.

### Statistical methods

Weighted differences of the means (MD) for continuous outcomes and their respective 95% confidence intervals (CI) were calculated for all studies included in the meta-analysis [24]. I2 index was used for the heterogeneity among the outcomes of different studies [25], with I2 ≥ 50% indicating significant heterogeneity [26]. Random effects model was applied [24]. Publication bias was tested by the Harbord-Egger’s test [27]. Categorical data were analysed with chi-square test and continuous data with Kruskal–Wallis test. Statistical significance was set at a p-level of 0.05. Meta-analysis was conducted using Review Manager (RevMan) for Mac (version 5.3. Copenhagen: The Nordic Cochrane Centre, The Cochrane Collaboration, 2014). The report of the study was complemented in adherence with the Preferred Reporting Items for Systematic Reviews and Meta-Analyses (PRISMA) group standards for reporting meta-analysis of observational studies [28].

### Ethics

No ethics board approval was needed as all original data were previously published.

## Results

**Fig. 2:**
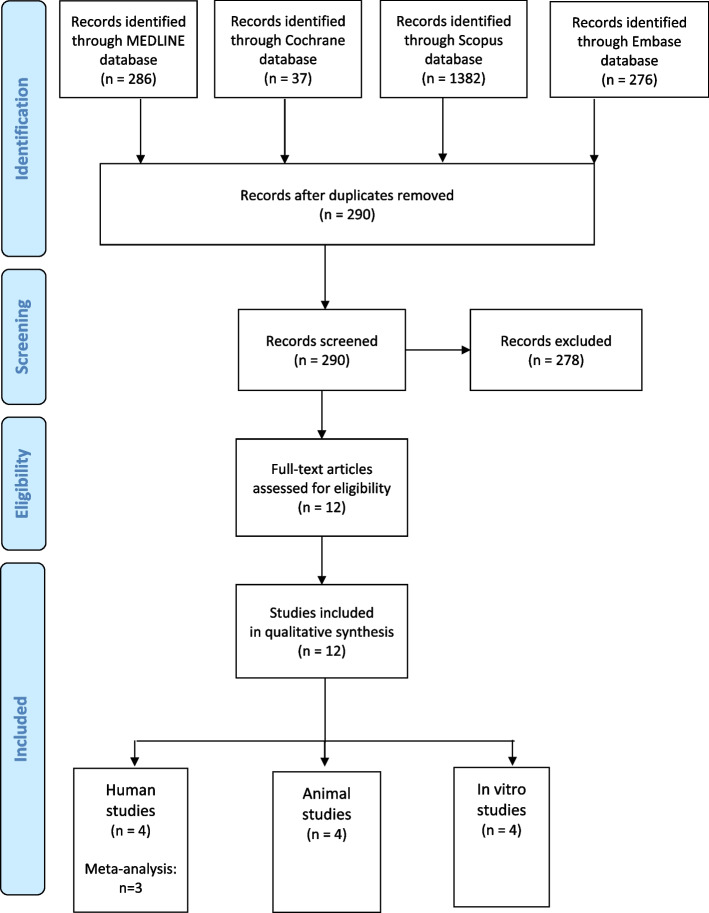
Study flow chart

After screening 290 identified studies, 278 studies were excluded by reading the abstracts. Finally, four human studies, four animal studies and four *in vitro* studies were included (Fig. 2).

### Human studies

The included four studies were randomized double blind trials comparing a group with vit D supplementation to placebo. Two of the above studies included women with surgically diagnosed endometriosis [29, 30], while the remaining studies included endometriosis patients whose diagnosis was based on their medical records [31, 32]. Groups in three of these studies consisted of women between 18 and 40 years old [29, 31, 32], while Nodler et al. [30] included young women between 12 and 25 years old.

Three studies examined the endometriosis-associated symptoms [29–31], while Somigliana et al*.* [32] examined fertility outcomes. Two studies were from Iran, one from the USA and one from Italy (Table 1).

**Table 1:** Clinical Trials examined the impact on Vit D supplementation in women with endometriosis

Study	Country	Treatment	Vit D (n)	Vit D endometriosis rASRM stage	Vit D age	Vit D group Vit D levels (ng/ml)	Placebo (n)	Placebo endometriosis rASRM stage	Placebo age	Placebo Vit D levels (ng/ml)	Conclusions
Almassinokiani et al., 2016 [29]	Iran	50 000 IU weekly for 12 weeks orally	19	I-II 11 III-IV 8	30.84 (5.79)	n/a	19 (20)	I-II 9 III-IV 10	28.95 (4.71)	n/a	After ablative surgery for endometriosis, vitamin D treatment did not have a significant effect in reducing dysmenorrhea (*p* = 0.45) and/or pelvic pain (*p* = 0.24)
Nodler et al., 2020 [30]	USA	2000 IU daily for 24 weeks orally	23 (27)	I-II 26 III-IV 1	20 (2.7)	Baseline 33.8 (4.5) 27 patients 6 months 42.5(4.8) 23 patients	19 (22)	I-II 21 III-IV 1	20.1 (3.5)	Baseline 31.2 (5.0) 22 patients 6 months 33.5(5.3) 19 patients	In young women with endometriosis, supplementation with vitamin D led to significant changes in pelvic pain (*p* = 0.02); however, these were similar in magnitude to placebo
Mehdizadehkashi et al., 2021 [31]	Iran	50 000 IU every two weeks for 12 weeks orally	25 (30)	n/a	34 (7.1)	Baseline 24.7 (7.6) 3 months 36.8 (8.1)	25 (30)	n/a	35.6 (7.0)	Baseline 25.4 (10.0) 3 months 25.9 (10.7)	Vitamin D intake in patients with endometriosis resulted in a significant improvement of pelvic pain, total-/HDL-cholesterol ratio, hs-CRP and TAC levels, but did not affect other clinical symptoms and metabolic profiles
Somigliana et al., 2021 [32]	Italy	single dose of 600,000 IU of vitamin D3	38	n/a	34.5 (2.5)	Baseline 21.8 (16.1–25.1)	45	n/a	34 (3)	19.7 (13.9–23.4)	In women with endometriosis, normal weight with preserved ovarian reserve and low vitamin D levels undergoing *in vitro* fertilization cycles, a single oral dose of 600,000 IU of vitamin D 3 did not improve the rate of clinical pregnancy

Vit D was supplemented orally in all studies, but dosage varied from 600,000 IU as single administration [32], to 50,000 IU weekly [29] or every two weeks [31] for 12 weeks, to 2000 IU daily for 24 weeks [30]. In addition, follow-up varied from 3 months [31] to 6 months [30]. All of the above included human studies had an overall low risk of bias as assessed with the Cochrane risk-of-bias tool for randomized studies (Supplementary Table 2).

In all of the above studies [30–32], except Almassinokiani et al. [29], randomization was conducted with computer generated random numbers, power analysis was performed and allocation concealment was used. In Almassinokiani et al. a simple randomization was performed and no information about power analysis and allocation concealement was reported [29].

#### Endometriosis-associated symptoms

Our systematic review includes 3 randomized controled trials with 231 women with endometriosis (114 women with vit D supplementation and 117 women with placebo). Endometriosis-associated pain was the primary outcome in all of the above studies [29, 30], except Mehdizadehkashi et al. [31], in which malondialdehyde was the primary outcome and the clinical symptoms were presented as secondary outcome. The VAS was used for the assessment of the pain in all of the included studies.

The Almassinokiani et al*.* [29] showed that a 50,000 IU vit D weekly supplementation for 12 weeks after ablative surgery for endometriosis did not have a significant effect on dysmenorrhea (*p* = 0.45) and pelvic pain (*p* = 0.24). Another study from Iran [31] with 50,000 IU vit D supplementation every two weeks for a total of 12 weeks showed a significant improvement of dysmenorrhea (*p* = 0.03), without any effect on dyspareunia or dyschezia. Nodler et al. [30] found a reduction of pelvic pain in young women with surgically diagnosed endometriosis after daily administration of 2,000 IU vit D for 24 weeks, which was not significant when compared to placebo (*p* = 0.97). The above study did not report outcomes on dysmenorrhea.

**Fig. 3:**
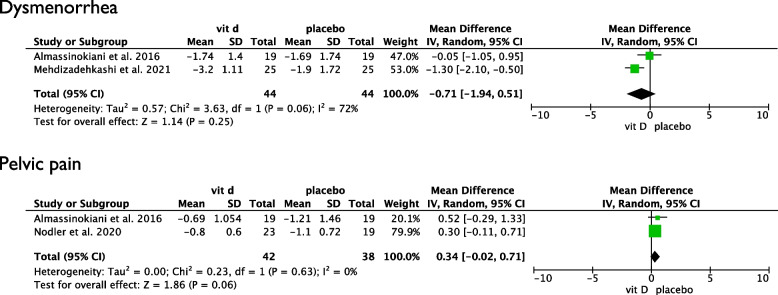
Quantitative synthesis results

The quantitative synthesis of the included studies showed no difference between placebo and vit D supplementation for dysmenorrhea (2 studies, 44 vit D vs 44 placebo, mean -0.71, 95% CI -1.94, 0.51) and non-cyclic pelvic pain (2 studies, 42 vit D vs 38 placebo, mean 0.34, 95% CI -0.02, 0.71) (Fig. 3). Assessment of publication bias is presented in Supplementary Material 4.

A subgroup analysis of women deficient in vit D (< 30 ng/ml) showed significantly lower intensity of dysmenorrhoea after vit D supplementation (1 study, 25 vit D vs 25 placebo, mean -1.30, 95% CI -2.10, -0.50) [31].

#### Fertility

The only available data for vit D supplementation in women with endometriosis aiming for pregnancy come from Somigliana et al. [32], who examined the effect of a 600,000 IU single dose of vit D in women with an initial serum level of 25(OH) D undergoing *in vitro* fertilization. In the endometriosis cohort (data of subgroup was provided upon communication with the corresponding author), the vit D group had similar cumulative pregnancy rate to the placebo group (*p* = 0.90) (38 patients with vit D, 45 patients with placebo). The estimated effect sizes for the duration of stimulation, the dosage of gonadotropins, oocytes retrieved and suitable oocytes were < 0.2, which indicates no significant difference.

### Animal studies

Four animal studies, published between 2012 and 2016 were included in our review. Three studies used mouse models with surgically induced endometriosis after performing endometrium auto-transplantation to peritoneum [33–35] and another study used a mouse model with injection of endometrium extracted from donor mice [36]. 2 studies used intraperitoneal administration of vit D [33, 34], one oral [36] and another intramuscular administration [35]. The quality assessment of the above studies with SYRCLE’s risk of bias tool showed an overall unclear risk of bias for all the included animal studies (Supplementary Table 3).

The results of Mariani et al., which showed reduction of endometriosis development and peritoneal inflammation after the administration of elocalcitol, a synthetic derivative of vit D [36], are in concordance with two other studies which also showed regression of endometriosis after treatment with vit D [34, 35]. On the other hand, Akyol et al. did not find any difference on regression of endometriosis implants after administration of vit D [33] (Table 2).

**Table 2:** Studies using mouse model of endometriosis to examine the effect of Vit D supplementation

Author	Country	Animal model	Study design	Intervention group	Control group	Dietary intervention	Results	Conclusions
Mariani et al., 2012 [36]	Italy	mouse model, endometriosis induced (allotransplantated endometrium) with injection	Controlled Trial	70	70	Elocalcitol (p.o.)	Elocalcitol led to ↓ lesion weight by 70% ↓ capacity of endometrial cells adherence to collagen inhibition of macrophage and inflammatory cytokine secretion	Elocalcitol inhibits endometriosis lesions development
Abbas et al., 2013 [34]	Jordan	mouse model, surgically induced endometriosis of autotrasplanted endometrium; 2 surgeries	Controlled Trial	9	8	Vit D (i.p.)	Vit D led to ↓ lesion by 48.8% ↑ fibrosis and apoptosis in stroma	Vit D reduces endometriosis lesions
Yildirim et al., 2014 [35]	Turkey	mouse model, surgically induced endometriosis of autotransplanted endometrium	Controlled Trial	7 simultaneous group 7 sequential group	7	1,25(OH)2D3 (i.m.)	1,25(OH)2D3 led to ↓ lesion volume, weight ↓ VEGF, MMP-9 levels ↑ TIMP-2	1,25(OH)2D3 regresses endometriotic implants in rat models by altering implant levels of VEGF, TIMP-2, and MMP-9
Akyol et al., 2015 [33]	Turkey	mouse model, surgically induced endometriosis of autotransplanted uterine horn	Controlled Trial	8 (Vit. D) 9 (Omega-3)	9	Vit. D (i.p.) Omega-3 (i.p.)	Vitamin D led to ↓ IL-6 levels	Omega-3 induced the regression of endometriosis implants in contrast to Vit D

### *In vitro* studies

Four *in vitro* studies exploring the effects of 1,25(OH)_2_D_3_ on human endometriotic stromal cells were identified. Miyashita et al. [13] examined the *in vitro* effects of 1,25(OH)_2_D_3_ on human endometriotic stromal cells, isolated from ovarian endometriomas. Use of 1,25(OH)_2_D_3_ reduced IL-1β-induced IL-8 mRNA expression (67.4 ± 9.4% vs. 72.1 ± 1.7%, *p* = 0.05), prostaglandin activity, viable endometrial stromal cell numbers and DNA synthesis, but it did not affect apoptosis when comparised to controls. Delbandi et al*.* [37] demonstrated that vit D increases cell adhesion and decreases invasion and proliferation of ectopic and eutopic endometrial stromal cells *in vitro* by reducing the production of IL-6, Bcl-2, Bcl-xL, and VEGF-α. Ingles et al*.* [38] showed that after treatment with vit D, endometriotic stroma cells have a higher CYP24A1 gene expression but decreased neuroangiogenesis, cellular motility and invasion. Yaghoubi et al. [39] found a significantly decreased expression of genes for EGF (epidermal growth factor), MDGF (monocyte/macrophage-derived growth factor) and PDGF (platelet-derived growth factor-B) in the endometriosis group but no effect in the control group when examining the impact of peritoneal fluid mononuclear cells exposure to vit D in women with and without endometriosis (Table 3).

**Table 3:** *In vitro* studies demonstrating the outcome of Vit D treatment of cells isolated from endometriosis lesions

Author	Country	Cell cultures	Assays used	Sample size	Treatment	Results
Delbandi et al., 2016 [37]	Iran	endometriotic stroma cells and eutopic endometrium	XTT proliferation assay, cell invasion assay, cell attachment assay, ELISA, RT-PCR, Western blot	45 (25 with endometrioma, 20 without endometriosis)	1,25(OH)2D3	↑ cell adhesion ↓ cell invasion, cell proliferation ↓ IL-6 and VEGF-A gene expression by ectopic stroma cells ↓ Bcl-2 gene expression by eutopic and Bcl-xL by ectopic stroma cells
Miyashita et al. 2016 [13]	Japan	endometriotic stroma cells isolated from ovarian endometrioma	RT-PCR, ELISA, enzyme immunoassay, cell-counting assay, bromodeoxzuridine incorporation assay, Western blot, RIA	35	1,25(OH)2D3	↓ IL-1β- or TNF-a-induced inflammatory responses ↓ endometriotic stromal cell numbers ↓ MMP-2 and MMP-9 ↓ DNA synthesis
Ingles et al., 2017 [38]	USA	endometriotic stroma cells isolated from peritoneal endometriosis lesions	RT-PCR	43 (38 endometriosis, 5 controls)	1,25(OH)2D3	↑ gene expression of CYP24A1 ↓ neuroangiogenesis ↓ cellular motility ↓ invasion
Yaghoubi et al., 2020 [39]	Iran	peritoneal fluid mononuclear cells	RT-PCR	20 (10 women with endometriosis, 10 women without endometriosis)	1,25(OH)2D3	Endometriosis ↓ gene expression of epidermal growth factor ↓ gene expression of platelet-derived growth factor-B ↓ gene expression of monocyte/macrophage-derived growth factor Non-endometriosis no significant effect on expression of above genes

## Discussion

In this meta-analysis of 4 randomized controlled trials with a total of 314 patients with endometriosis, vit D supplementation was neither significantly associated with pelvic pain or dysmenorrhea amelioration nor did it improve fertility outcomes. No meta-analyses on this topic have been previously published.

In women with primary dysmenorrhea without the diagnosis of endometriosis different dosages (single 300.000 IU dose 5 days before menstruation [40, 41] or 50.000 IU/ weekly [42, 43]) of vit D supplementation can reduce pain intensity. A significantly greater mean decrease in pain has been also observed in a systematic meta-analysis evaluating vit D supplementation in patients with different kinds of pain (musculosceletal pain, arthritis, dysmenorrhea, migraine) xxx (mean difference -0.57, 95% CI: -1.00 to -0.15, *P* = 0.007) [44]. However, prior to our review it was unclear whether women diagnosed with endometriosis also benefit from vit D supplementation.

The only available randomized controlled study on vit D supplementation in a group of women with endometriosis receiving IVF did not show any significant differences for reproductive outcomes [32]. Perhaps this outcome is not surprising as previous studies in patients without the diagnosis of endometriosis showed discrepancies in the effect of vit D before ART on reproductive outcomes. Specifically, vit D supplementation of 50.000 IU weekly for 6 weeks before an embryo transfer was not associated with a significant improvement of pregnancy rates [45], while the same dose for 6 to 8 weeks was related to higher clinical pregnancy rate after ICSI in another study [46]. Interestingly, vit D in combination with different additional substances (vit E, folic acid, alpha-lactalalbumin, myo-inositol, melatonin, omega-3 and olive oil) showed some benefits in the context of IVF treatment [47–49].

A key observation that led to our metaanalysis is that vit D insufficiency is associated with pain severity in patients with endometriosis [11]. The mean baseline vit D concentration was higher than 30 ng/ml in Nodler et al*.* [30], but below this threshold in Mehdizadehkashi et al*.* [31] and Somigliana et al*.* [32]. Baseline concentrations are not reported in Almassinokiani et al*.* [29]. The variability regarding the status of vit D deficiency in the analysed cohorts may partly explain the discrepancies in findings. VDR polymorphisms, which play an important role in the bioavailability of vit D [50] were not assessed in the included studies and might be another reason for the different outcomes.

The characteristics of some host-related factors between the participants of the included studies which affect the metabolism and hence absorption of vit D are additional contributors to the results and should be carefully considered. For example, the mean age of the participants varied from 20 [30] to 35.6 years [31]. Back in 1978, a lower response after oral supplementation in older women in comparison to younger women was reported [50], suggesting an inadequate absorption of cholecalciferol in the older population. Mean BMI varied between 22.46 [29] and 26.2 [30] kg/m^2^, which could lead to lower levels of vit D due to volumetric dilution in individuals with higher BMI [51]. Gastrointestinal diseases and bariatric surgery procedures, which reduce the absorption of vit D [52], were addressed as exclusion criteria only in Nodler et al*.* [30].

Endometriosis heterogeneity including severity (rASRM stage of disease) and phenotypes (peritoneal endometriosis, deep infiltrating endometriosis and ovarian endometriosis) may further explain discrepancies in pathophysiology and clinical manifestation [1]. The majority of the patients in Almassinokiani et al*.* [29] had rASRM stage III-IV disease but rASRM stage I-II in Nodler et al*.* [30], while in Mehdizadehkashi et al*.* [31] the rASRM stage was not reported. The different phenotypes of endometriosis were not specified in any of the included studies. Furthermore, additional hormonal treatments during or before the vit D supplementation period were not clearly described in the included studies. Only Almassinokiani et al*.* [29] reported that patients had a laparoscopy 24 weeks before the vit D treatment so that it is unclear if the other included studies refer to a similar study population.

Animal models have shown a significant reduction of the lesions’ size [34–36], possibly explained by decreased cellular motility, proliferation, and invasion [37, 38]. Unfortunately, in endometriosis research the results of animal and *in vitro* models are often inconsistent with the results of human studies [53]. These discrepancies are probably related to the differences between humans and animal/ *in vitro* models, the multifactorial nature of endometriosis and the complicated metrics of drug efficacy.

Other human studies have evaluated molecular or histological outcomes after vit D supplementation. A recent *in vivo* study examining CD44 expression (a protein that seems to play a role on cell adhesion) in endometrial cells of women with endometriosis showed a significant decrease after administration of oral vit D [54]. Fibrosis, which seems to play a role in endometriosis has also been shown to be reduced after vit D treatment in histological samples [34]. This could be partially explained by the reduced expression of matrix metalloproteinases 2 and 9 (MMP-2, -9) [13, 35]. Finally, another beneficial impact of vit D supplementation could be a reduction of inflammation on various levels, including distinct pathways suppression [13, 37], decreased cytokine secretion [33, 35, 36] and macrophage recruitment [36].

A limitation of the review is the low number of included studies in the meta-analysis as well as their small sample size, so that the statistical power might have been inadequate to identify potential significant associations between vit D supplementation and study outcomes. The heterogeneity of the included studies regarding the baseline characteristics (age, BMI and vit D concentrations) and vit D dosages, as discussed above, represents another study limitation. Regardless of these limitations, it is pivotal to provide the scientific community with all available evidence, that can help guide clinical practice and inspire further research contributing to the unravelling of the question whether vit D supplementation can improve symptoms associated with endometriosis.

## Conclusion

Although *in vitro* and animal studies seem to suggest regression of the endometriotic implants and decrease of invasion and proliferation after vit D supplementation, this was not reflected in the results of the included human studies. This review and meta-analysis showed that vit D supplementation in patients with endometriosis seems not to have a clinical effect on the improvement of dysmenorrhea or non-cyclic pelvic pain or IVF outcomes. However, given the heterogeneity and the diversity of the available studies, more research is required to shed light on the role of vit D supplementation in women with endometriosis. Vit D supplementation should be considered to be used in women with low levels of vit D for protection against conditions related to vit D deficiency, such as osteoporosis.

## Supplementary Information

**Additional file 1: Supplementary material 1.** Summary of *in vitro *and *in vivo* supplementation studies.

**Additional file 2: Supplementary Table 2**. Quality assessment of RCT human studies.

**Additional file 3: Supplementary Table 3.** Quality assessment of animal studies (SYRCLE tool).

**Additional file 4: Supplementary material 4.** Funnel plots.

## Data Availability

The datasets supporting the conclusions of this article are included within the article and its additional file.
