# Supplementary material for: Effects of vitamin D supplementation in endometriosis: a systematic review
Source: Reprod Biol Endocrinol. 2022 Dec 28;20:176. doi: 10.1186/s12958-022-01051-9 (PMC9795583; doi:10.1186/s12958-022-01051-9)
Supplement: Supplementary file 1 — Additional file 1: Supplementary material 1. Summary of in vitro and in vivo supplementation studies. [file 12958_2022_1051_MOESM1_ESM.docx]

| **In vitro** | **Animal studies** | **Clinical Studies** |
| --- | --- | --- |
| ↑ gene expression of CYP24A1, ↓ neuro-angiogenesis, cellular motility, and invasion pathways | ↓ endometriotic implants’ cyst cross sectional area - fibrosis and apoptosis in the stroma | after surgical treatment no significant effect in reducing dysmenorrhea and/or pelvic pain |
| ↓ IL-1β and TNF-α-induced inflammatory responses: IL-8 expression and prostaglandin activity - ↓ viable ESC numbers and DNA synthesis - ↓ MMP-2 and MMP-9 expressions and inhibited nuclear factor-κB activation - did not affect apoptosis | ↓ total lesion weight, endometrial cells adherence to collagen, macrophage recruitment, and inflammatory cytokine secretion | improvement of pelvic pain, total-/HDL-cholesterol ratio, hs-CRP and TAC levels |
| ↑ cell adhesion, ↓ invasion and proliferation of ectopic and eutopic endometrial stromal cells - ↓ IL-6, Bcl-2, Bcl-xL, and VEGF-α production | ↓ endometriotic implants’ histologic score, mean volume, and weight - ↓ VEGF, MMP–9 levels - ↑ TIMP–2 levels | significant changes in pelvic pain; however, these were similar in magnitude to placebo |
| ↓ gene expression of epidermal growth factor  ↓ gene expression of platelet-derived growth factor-B  ↓ gene expression of monocyte/macrophage-derived growth factor | ↓ IL-6 levels | examined fertility outcomes; same cumulative pregnancy rate in comparison to placebo |
|  |  | ↓ expression level of CD44s, CD44V, and CD44v6 in the eutopic endometrium as well as the concentration of soluble CD44 in the endometrial fluid |

# **Supplementary material 1:** Summary of *in vitro* and *in vivo* supplementation studies
