# Supplementary material for: Effects of vitamin D supplementation in endometriosis: a systematic review
Source: Reprod Biol Endocrinol. 2022 Dec 28;20:176. doi: 10.1186/s12958-022-01051-9 (PMC9795583; doi:10.1186/s12958-022-01051-9)
Supplement: Supplementary file 2 — Additional file 2: Supplementary Table 2. Quality assessment of RCT human studies. [file 12958_2022_1051_MOESM2_ESM.docx]

|  | Random sequence generation (selection bias) | Allocation concealment (selection bias) | Blinding of participants and personnel (performance bias) | Blinding of outcome assessment (detection bias) | Incomplete outcome data (attrition bias) | Selective reporting (reporting bias) |
| --- | --- | --- | --- | --- | --- | --- |
| Almassinokiani et al. 2016 [29] | Low | Low | Low | Low | Low | Low |
| Nodler et al., 2020 [31] | Low | Low | Low | Unclear | Low | Low |
| Mehdizadehkashi et al. 2021 [30] | Low | Low | Low | Low | Low | Unclear |
| Somigliana et al., 2021 [32] | Low | Low | Low | Low | Low | Low |

**Supplementary Table 2**: Quality assessment of RCT human studies
