# Supplementary material for: Effects of vitamin D supplementation in endometriosis: a systematic review
Source: Reprod Biol Endocrinol. 2022 Dec 28;20:176. doi: 10.1186/s12958-022-01051-9 (PMC9795583; doi:10.1186/s12958-022-01051-9)
Supplement: Supplementary file 3 — Additional file 3: Supplementary Table 3. Quality assessment of animal studies (SYRCLE tool). [file 12958_2022_1051_MOESM3_ESM.docx]

**Supplementary Table 3**: Quality assessment of animal studies (SYRCLE tool)

|  | Sequence generation (selection) | Baseline characteristic (selection) | Allocation concealment (selection) | Random housing (performance) | Blinding (performance) | Random outcome assessment (detection | Blinding (detection) | Incomplete outcome data (attrition) | Selective outcome reporting (reporting) | Other biases |
| --- | --- | --- | --- | --- | --- | --- | --- | --- | --- | --- |
| Mariani et al., 2012 [36] | U | L | U | U | U | U | U | L | L | L |
| Abbas et al., 2013 [34] | U | L | U | U | U | U | U | U | L | L |
| Yildirim et al., 2014 [35] | U | L | U | L | U | U | U | L | L | L |
| Akyol et al., 2015 [33] | U | L | U | L | U | U | U | L | L | L |

L, low risk; H, high risk; U, unclear risk
