# Supplementary figures and images for: Effects of vitamin D supplementation in endometriosis: a systematic review
Source: Reprod Biol Endocrinol. 2022 Dec 28;20:176. doi: 10.1186/s12958-022-01051-9 (PMC9795583; doi:10.1186/s12958-022-01051-9)

**Supplementary material 4.** Funnel plots

Dysmenorrhea


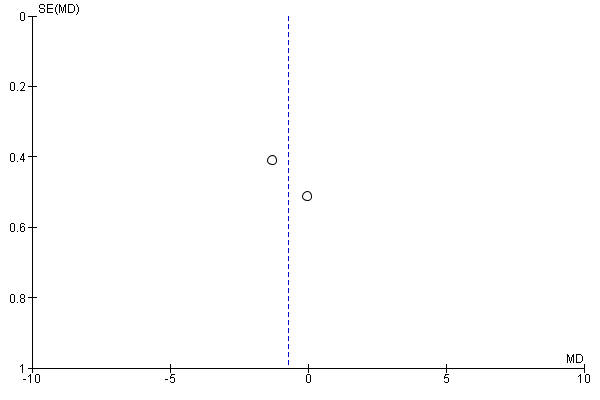


Pelvic Pain


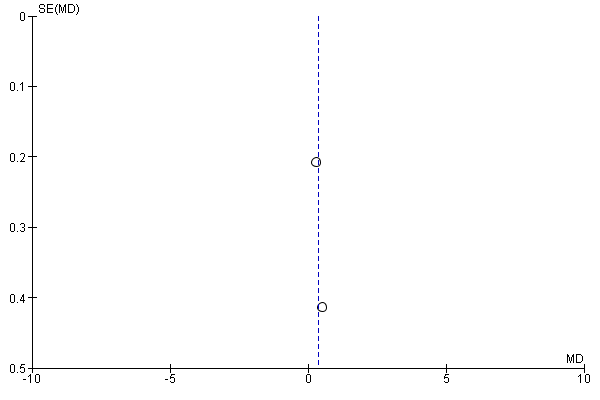

Supplement: Supplementary file 4 — Additional file 4: Supplementary material 4. Funnel plots. [file 12958_2022_1051_MOESM4_ESM.docx]
